# Supplementary material for: Host E3 ubiquitin ligase ITCH mediates Toxoplasma gondii effector GRA35-triggered NLRP1 inflammasome activation and cell-autonomous immunity
Source: mBio. 2024 Feb 20;15(3):e03302-23. doi: 10.1128/mbio.03302-23 (PMC10936166; doi:10.1128/mbio.03302-23)
Supplement: Supplemental Figures — Figures S1 to S5 [file mbio.03302-23-s0001.docx]

**Table S1**

This table contains the raw sgRNA reads and MAGeCK analysis results for each CRISPR screen.

**Table S2**

Primers and oligos used in this study.

**Table S3**

Mass spectrometry data with unique spectrum count for each immunoprecipitated sample.

**Figure S1**

**
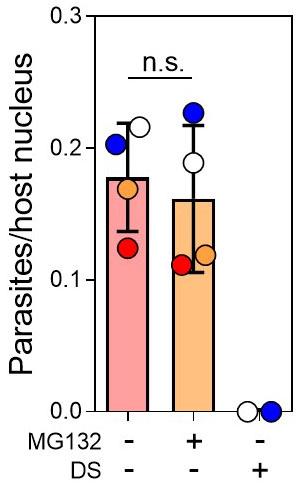
**

**Figure S1 Parasite invasion in MG132-treated Lewis rat BMDMs.**

Lewis rat BMDMs pre-treated with 0.5 μM MG132, 30 mg/ml dextran sulfate (DS), or left untreated for 2 h were infected with *Toxoplasma* parasites for 30 mins. The parasite invasion rate in each media was measured by red/green invasion assay and is expressed as a ratio of invaded parasites vs. number of host nuclei. Data are displayed as mean ± SD with independent experiments (*n* = 4) indicated by the same color dots. Significance was determined with one-way ANOVA with Tukey’s multiple comparisons test.

**Figure S2**


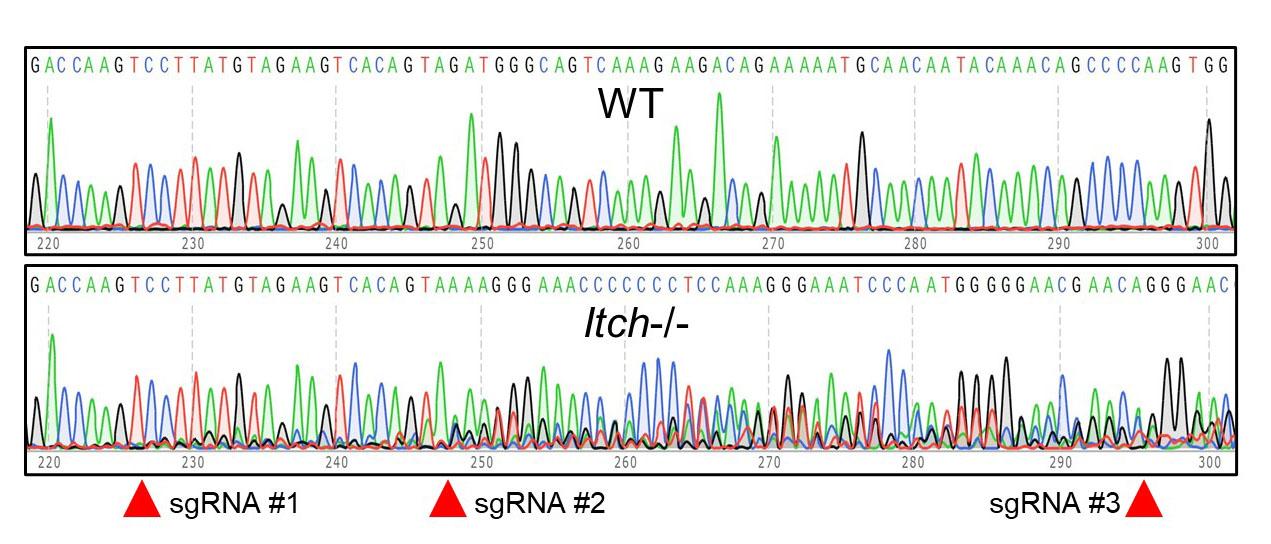


**Figure S2**

Sequencing chromatogram of the *Itch* sgRNA targeting region in Lewis rat BMDMs transfected with Cas9 protein only (WT) or Cas9 protein assembled with Itch sgRNAs (*Itch*-/-). Red arrowheads indicate the CRISPR/Cas9 cleavage sites.

**Figure S3**

**
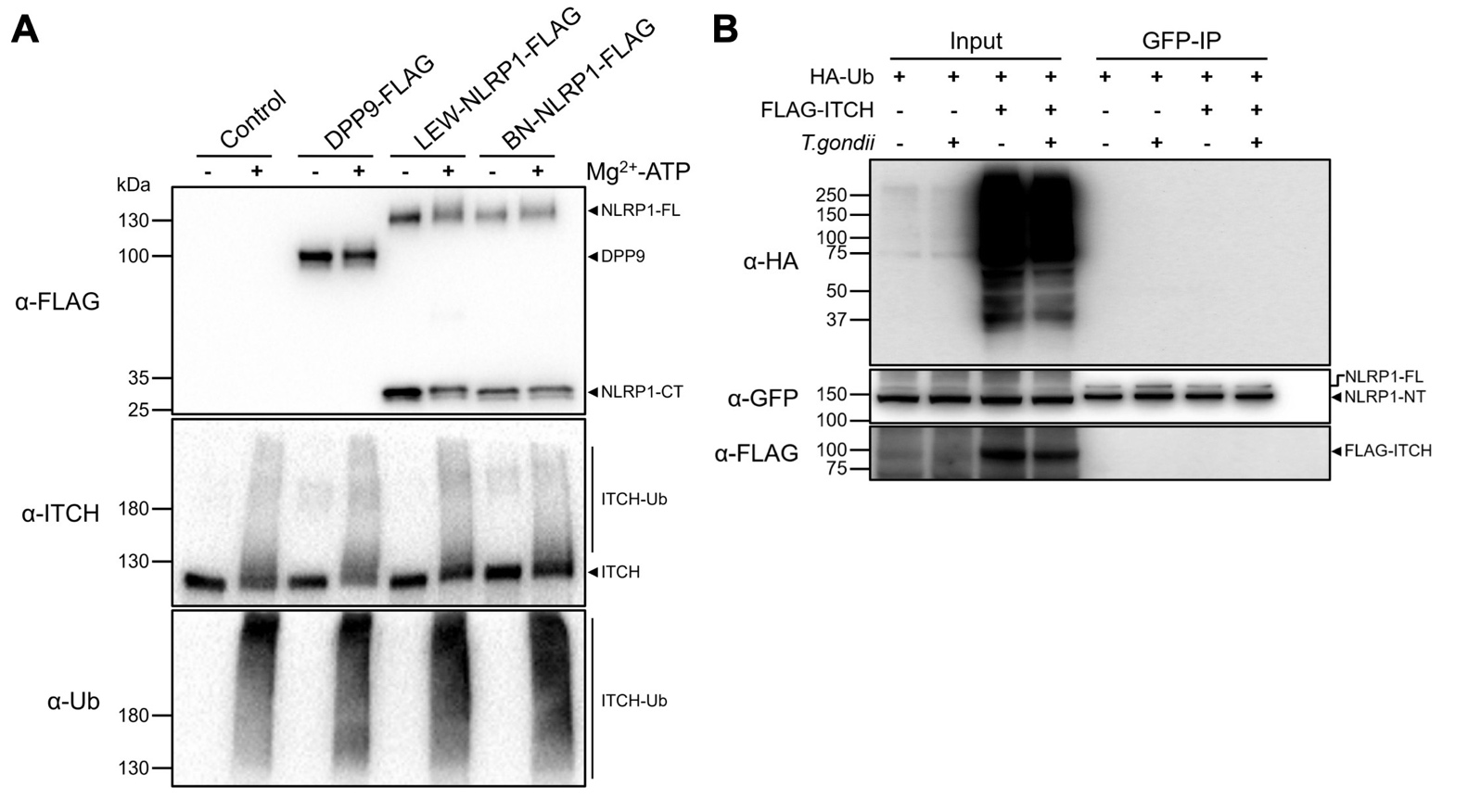
**

**Figure S3 ITCH neither directly ubiquitinates nor interacts with Lewis rat NLRP1.**

**A**. In vitro ubiquitination assay using purified recombinant E1, ubiquitin-conjugating enzyme UBE2L3, human ITCH, and rat DPP9 or rat NLRP1 proteins, together with Ubiquitin and ATP. The mixture was incubated for 2h at 37 °C, quenched by the addition of SDS loading buffer, and analyzed by SDS-PAGE and immunoblotting with indicated antibodies. The images are representative of results from 2 independent experiments.

**B**. HEK293T cells stably expressing EGFP-NLRP1-MYC were transfected with HA-Ubiquitin (HA-Ub) with or without FLAG-rITCH. At 24 h post-transfection, the cells were treated with 5 μM MG132 for 2 h followed by infection with *Toxoplasma* parasites (MOI = 2) or left uninfected for another 6 h. NLRP1 was immunoprecipitated from the cell lysate using GFP antibodies, and its ubiquitination and ITCH presence were detected with HA and FLAG antibodies, respectively. 5% of the total lysate was loaded and used as input. The images are representative of results from 2 independent experiments.

**Figure S4**

**
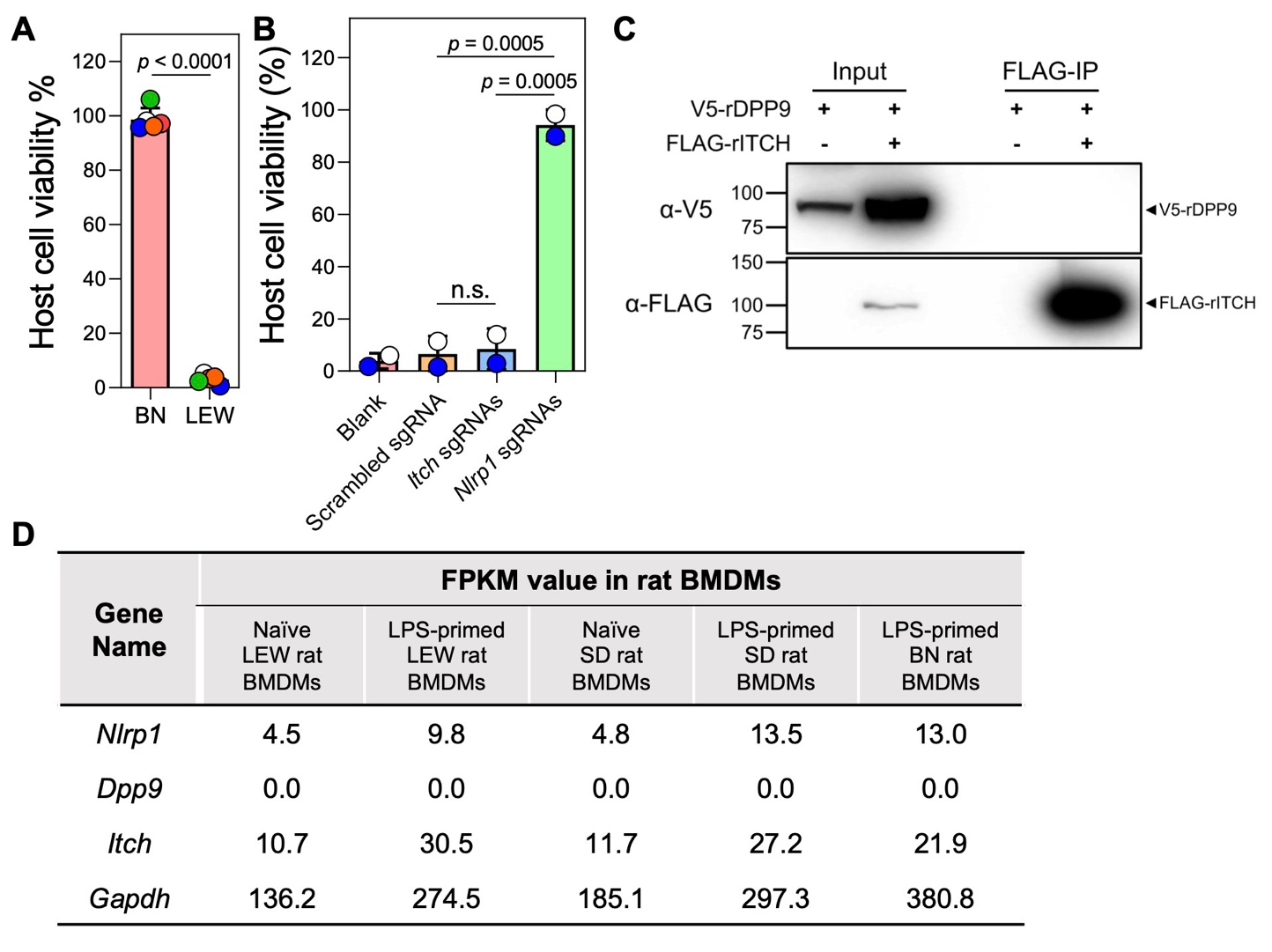
**

**Figure S4 VbP-induced activation of the Lewis rat NLRP1 inflammasome is independent of ITCH.**

**A**. Brown Norway (BN) or Lewis (LEW) rat BMDMs were incubated with VbP (2 μM) for 24 h. Macrophage viability was measured via MTS assay. Data are displayed as mean ± SD with independent experiments (*n* = 5) indicated by the same color dots. Significance was determined with two-tailed paired *t*-test.

**B**. Indicated Lewis rat BMDMs were incubated with VbP (2 μM) for 24 h. Macrophage viability was measured via MTS assay. Data are displayed as mean ± SD with independent experiments (*n* = 2) indicated by the same color dots. Significance was determined with one-way ANOVA with Tukey’s multiple comparisons test.

**C**. Lysates of HEK293T cells transiently expressing FLAG-ITCH and V-tagged DPP9 were immunoprecipitated using FLAG antibodies and analyzed by immunoblotting with indicated antibodies. 5% of the total lysate was loaded and used as input. The images are representative of results from 2 independent experiments.

**D**. The FPKM value of indicated genes determined from our previous transcriptomic analysis^7^ in naïve or LPS-primed rat BMDMs is shown. SD rat indicates Sprague Dawley rat.

**Figure S5**


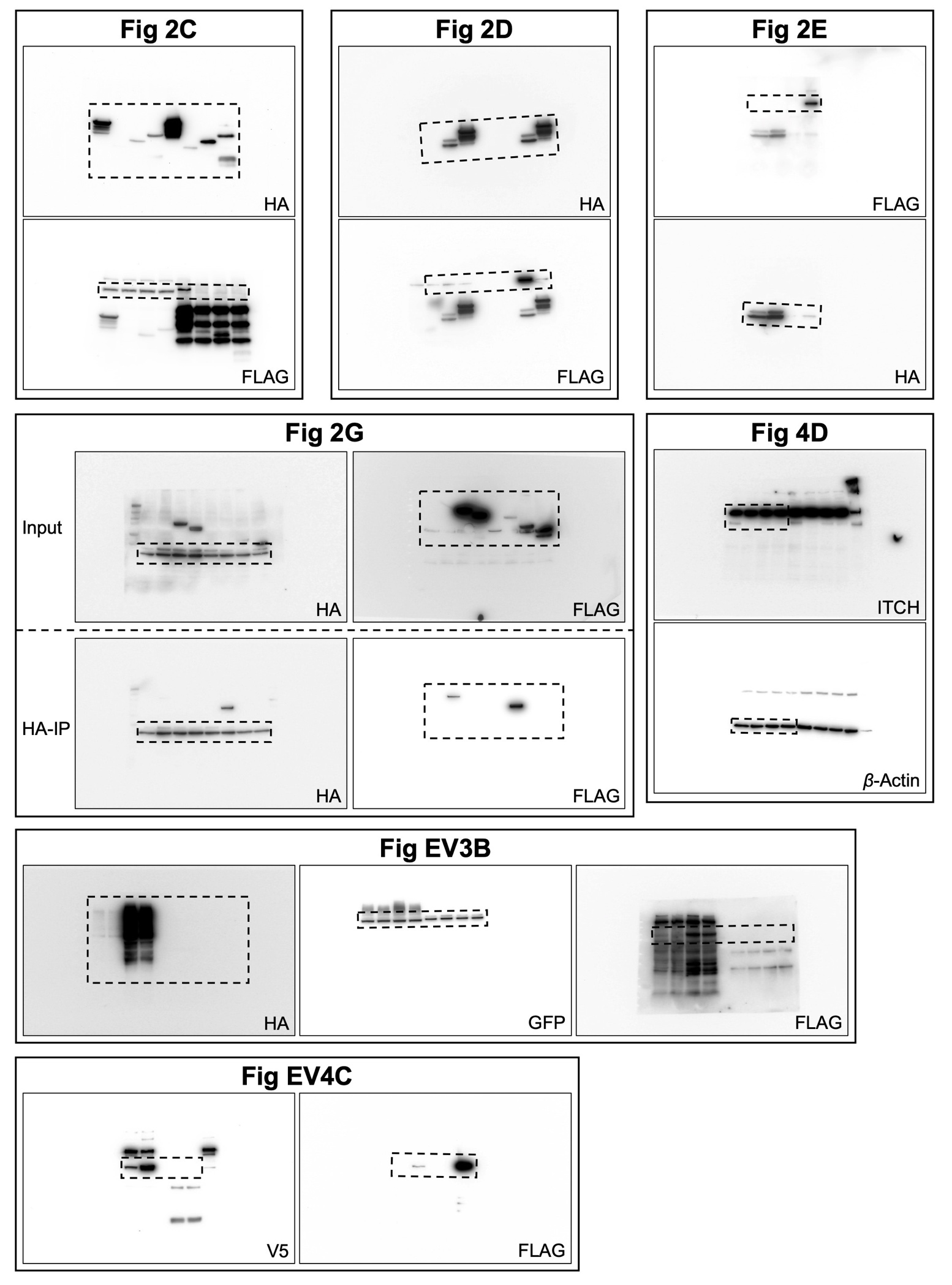


**Figure S5 All uncropped Western blot images in this study.**
