# Supplementary material for: Host E3 ubiquitin ligase ITCH mediates Toxoplasma gondii effector GRA35-triggered NLRP1 inflammasome activation and cell-autonomous immunity
Source: mBio. 2024 Feb 20;15(3):e03302-23. doi: 10.1128/mbio.03302-23 (PMC10936166; doi:10.1128/mbio.03302-23)
Supplement: Description of Supplemental Tables — Full supplemental table legends. [file mbio.03302-23-s0003.docx]

**Description of Supplementary Tables**

**Table S1 Description:**

Sheet 1 “Raw reads”, this tab contains the raw sgRNA reads for each CRISPR screen.

Sheet 2 “Positive enrichment”, this tab contains the *p*-value and number of enriched sgRNAs for each gene analyzed by the MAGeCK algorithm.

**Table S2 Description:**

Sheet 1 “Plasmids”, this tab contains all the plasmids generated in this study.

Sheet 2 “Primers for plasmid generation”, this tab contains all the primers used for constructing the plasmids.

Sheet 3 “Primers for Illumina sequencing”, this tab contains all the primers used for the Illumina sequencing

Sheet 4 “sgRNA sequence”, this tab contains all the sgRNA targeting sequences used in this study.

**Table S3 Description:**

Sheet 1 “Exp.1”, this tab contains all the total unique spectrum counts for proteins identified via Mass Spectrometry in the first independent GRA35-IP experiment.

Sheet 2 “Exp.2”, this tab contains all the total unique spectrum counts for proteins identified via Mass Spectrometry in the second independent GRA35-IP experiment.

Sheet 3 “(Mukhopadhyay et al., 2020)”, this tab contains all the total unique spectrum counts for proteins identified via Mass Spectrometry in our published study (Mukhopadhyay et al., 2020).
